# NADK tetramer defective mutants affect lung cancer response to chemotherapy via controlling NADK activity

**DOI:** 10.1016/j.gendis.2024.101510

**Published:** 2025-01-07

**Authors:** Mengxue Hu, Fuxing Wang, Yue Zhu, Yi Yao, Huadong Pei, Zheng Liu, Pingfeng Zhang

**Affiliations:** a Cancer Center, Renmin Hospital of Wuhan University, Wuhan, Hubei 430060, China; b School of Medicine, Kobilka Institute of Innovative Drug Discovery, The Chinese University of Hong Kong (Shenzhen), Shenzhen, Guangdong 518172, China; c Department of Radiotherapy, The First Affiliated Hospital, Medical School of Xi'an Jiaotong University, Xi'an, Shaanxi 710061, China; d Department of Oncology, Georgetown Lombardi Comprehensive Cancer Center, Georgetown University Medical Center, Washington, DC 20057, USA

**Keywords:** Chemotherapy resistance, Lung cancer, Methylation, NADK mutants, NADK tetramer

## Abstract

Nicotinamide adenine dinucleotide (NAD^+^) kinase (NADK) phosphorylates NAD^+^ to generate NADP^+^, which plays a crucial role in maintaining NAD^+^/NADP^+^ homeostasis, cellular redox balance, and metabolism. However, how human NADK activity is regulated, and how dysregulation or mutation of NADK is linked to human diseases, such as cancers, are still not fully understood. Here, we present a cryo-EM structure of human tetrameric NADK and elaborate on the necessity of the NADK tetramer for its activity. The N-terminal region of human NADK, which does not exist in bacterial NADKs, modulates tetramer conformation, thereby regulating its activity. A methylation-deficient mutant, R45H, within the N-terminal region results in increased NADK activity and confers cancer chemotherapy resistance. Conversely, mutations in NADK identified among cancer patients alter the tetramer conformation, resulting in NADK inactivation and increasing the sensitivity of lung cancer cells to chemotherapy. Our findings partially unveil the structural basis for NADK regulation, offering insights into the cancer etiology of patients carrying NADK mutations.

## Introduction

NAD(H) and NADP(H) serve as crucial electron carriers in cellular metabolism, playing distinct roles in catabolism and anabolism, respectively.^1^^,^^2^ They also act as essential cofactors for a multitude of enzymes involved in various biological processes.^3^^,^^4^ NAD^+^ kinase (NADK) is the key regulator of cellular NAD^+^/NADP^+^ homeostasis, which catalyzes the conversion of NAD^+^ to NADP^+^.^5^^,^^6^ NADK is pivotal for numerous cellular functions, including metabolism, energy production, DNA repair, cell signaling, and more.^7^, ^8^, ^9^ Despite its importance, the detailed structure basis of enzymatic catalysis and the regulatory mechanisms of NADK remain incompletely understood. Particularly, the regulatory roles of the mammalian NADK, which has evolved an additional N-terminal compared with the bacterial NADK, remain elusive.^10^

Highly proliferating cancer cells require ample quantities of the reducing agents NADH and NADPH to fuel energy production and drive the reductive synthesis of biomaterials.^11^^,^^12^ However, this heightened metabolic activity also increases the production of reactive oxygen species (ROS), causing damage to cellular components and promoting cancer progression.^13^ Additionally, all anti-neoplastic agents induce ROS by diverting the electron transport system, further exacerbating ROS levels in cancer cells.^14^^,^^15^ Consequently, cancer cells usually possess high levels of ROS and need to manage oxidative stress. NADPH plays a critical role in counteracting the impact of ROS, maintaining cellular redox status.^11^^,^^16^ Perturbations in NADK, such as alterations in protein expression or enzymatic activity, can disturb cellular NAD^+^/NADP^+^ levels, thereby influencing ROS levels and potentially promoting cancer progression and metastasis.^17^^,^^18^

Since NADP^+^ is impermeable to the cell membrane, mammalian cells possess distinct cytoplasmic NADK (cNADK) and mitochondrial NADK (mNADK) to regulate NADP^+^ levels in each cellular compartment. mNADK harbors a mitochondrial localization signaling peptide and determines the mitochondrial NADP(H) levels, forming a dimer that differs from cNADK and has different regulatory mechanisms.^19^^,^^20^ As mNADK deficiency causes significant metabolic and developmental abnormalities,^21^ its critical role in vital organs like the heart and brain limits its clinical application.^21^^,^^22^ Consequently, directing therapeutic attention towards cNADK emerges as a more viable strategy for cancer treatment.^17^^,^^18^

Although the mechanisms by which cancer cells regulate cNADK levels remain unclear, certain gain-of-function mutants in cNADK have been associated with increased NADPH levels and reduced ROS levels. For instance, the I90F mutant has been implicated in driving pancreatic tumor progression.^23^ Meanwhile, many cNADK mutations occur in cancer patients, but their roles and mechanisms in cancer development and treatment response are unclear. More recently, phosphorylation in the N-terminal regulatory domain has been identified to activate NADK by relieving an auto-inhibitory conformation.^18^^,^^24^^,^^25^ Our work also revealed that methylation in this N-terminal domain inhibits NADK activity (side-by-side submission). A comprehensive understanding of how the N-terminal region of cNADK and various cancer-associated mutants regulate its function may elucidate cancer development and conquer chemotherapy resistance.

In this study, we investigated the regulatory mechanism of cNADK by determining the cryo-electron microscopy (cryo-EM) structure of the cNADK tetramer. We also characterized the effects of cancer-associated NADK mutants on NADK activity and cancer cell response to chemotherapy. This work therefore reveals molecular insights into NADK dysregulation in cancer and could have a substantial translational impact on the diagnosis and targeted treatment of NADK-associated cancers.

## Materials and methods

### Antibodies

Rabbit against human NADK monoclonal antibody (CST, #89833, 1:1000); mouse against human beta-tubulin monoclonal antibody (Proteintech, #66240–1-Ig, 1:5000); mouse against HA tag antibody (Sigma, #H9658, 1:3000); and rabbit against Mono-Methyl Arginine [α-mme-R] (CST, #8015, 1:1000) were used.

### Plasmids

Human NADK was amplified through PCR and cloned to modified pET28a, pCMV-HA, and pCDH-FLAG vectors. NADK variants were generated by site-directed mutagenesis and confirmed by sequencing. The sgRNA oligonucleotides targeting human NADK were cloned into a Lenti CRISPR V2 vector. The targeting sequences were as follows: sense, AGTTACAACCACCCCATCCG; antisense, TCAATGTTGGTGGGGTAGGC.

### Cell culture

HEK293T cells were obtained from the American Type Culture Collection (ATCC, USA) and cultured in high-glucose Dulbecco's modified Eagle's medium supplemented with 10% fetal bovine serum and 1% penicillin-streptomycin. H520 cells were kindly provided by the National Center for Protein Sciences and cultured in Roswell Park Memorial Institute (RPMI) 1640 medium supplemented with 10% fetal bovine serum and 1% penicillin-streptomycin. All cells were cultured at 37 °C in a 5% (*v*/*v*) CO_2_ incubator.

### Mice

Male BALB/c nude mice (6 weeks old) were purchased from Wuhan Moubaili Biotechnology Co., Ltd., China, and raised in a specific pathogen-free environment at the Hubei Food and Drug Safety Evaluation Center (China). The Institutional Animal Care and Use Committee approved all the mouse experiments.

### Expression and purification of human NADK

The prokaryotic vector plasmids of human NADK were transformed into *E. coli* BL21 cells. A single colony strain was selected for overnight culture and then transferred to 1L sterilized LB medium containing 50 μg/mL kanamycin and cultured on a shaker at 37 °C. When the optical density at 600 nm (OD600) reached between 0.6 and 0.8, isopropyl β-d-1-thiogalactopyranoside (IPTG) was added to a final concentration of 0.1 mM and cultured overnight at 18 °C to induce overexpression of NADK protein. The cells were harvested by centrifugation at 4 °C and disrupted using a high-pressure cell disruptor in buffer containing 50 mM Tris–HCl (pH 7.5) and 500 mM NaCl supplemented with 1 mM phenylmethyl sulfonyl fluoride (PMSF) and complete protease inhibitor cocktail. After centrifugation, the supernatant was loaded onto a HisTrap FF column (GE Healthcare). The human NADK protein was eluted by competitive imidazole binding. The eluted NADK protein was incubated overnight with TEV protease to cleave off the His tag. The resulting mixture was loaded onto a resource Q ion exchange column (GE Healthcare) to remove the contaminant proteins. Subsequently, the purified NADK protein was collected and loaded onto a Superdex 200 Increase 10/300 GL column (GE Healthcare). The tetramer of NADK protein was collected based on the elution volume and concentrated to a protein concentration of 10 mg/mL. The samples were aliquoted and flash-frozen in liquid nitrogen or used for cryo-EM studies immediately.

### Cryo-grids preparation and data collection

To prepare cryo-EM sample grids, 3 μL aliquots of the NADK tetramer sample (10 mg/mL) were applied onto glow-discharged holey carbon copper grids (Quantifoil Au R1.2/1.3, 300 mesh). After incubating the grids at 4 °C under 100% humidity for 10 s, they were blotted for 3.0 s and then plunge-frozen into liquid ethane cooled by liquid nitrogen using a Vitrobot (Mark IV, Thermo Fisher Scientific). Cryo-EM datasets were acquired on the Titan Krios microscope operating at 300 kV, equipped with a Gatan K3 Summit detector and a GIF Quantum energy filter with a 20 eV slit width. Images were recorded with SerialEM, with magnification at 105 K and a pixel size of 0.83 Å. Movie stacks were automatically acquired in super-resolution mode with 2-time hardware binning (105,000 × magnification) using SerialEM, with a defocus range from −1.0 μm to −2.0 μm. Each stack was exposed for 2.5 s with 0.05 s per frame, resulting in 50 frames and a total dose of approximately 22 e−/pixel/s.

### Structure determination and refinement

The image processing employed here follows a hierarchical approach, as described in previous studies.^26^^,^^27^ Initially, raw movie frames were aligned with MotionCor2 with a 9 × 7 patch, and contrast transfer function (CTF) parameters were estimated using Gctf and ctf in JSPR.^28^ Micrographs with consistent CTF values, including defocus and astigmatism parameters, were selected for further image processing. Data binned 4 times were utilized for micrograph screening and particle picking, while data with 2-time binning were employed for particle screening and classification. Following initial cleaning, particles were extracted using the cleaned micrographs, and the resulting dataset underwent final cleaning and reconstruction. Resolution estimates were obtained by applying a soft mask around the protein density and utilizing the gold-standard FSC = 0.413 criterion. The local resolution map was calculated using ResMap.^29^ The NADK resolution map at 2.54 Å was used for complete model building. The crystal structure of the NADK (pdb code 3PFN) served as the initial model. It was docked into the cryo-EM map and manually adjusted in coot.^30^ The cryo-EM electronic density map facilitated the construction of a model of tetrameric NADK. All structure refinements were conducted using PHENIX.^31^ Structure figures were generated using PyMOL (http://www. pymol.org) and Chimera.^32^

### Cross-linking

After washed with ice-cold phosphate buffer saline solution (PBS) three times, the cells were collected and resuspended in PBS supplemented with 1 mM PMSF and a complete protease inhibitor cocktail. The cells were disrupted by sonication for 30 s and centrifuged at 13,000 *g* and 4 °C for 10 min. The supernatant was collected for cross-linking experiments. BS3 (ThermoFisher, #A39266) was added to the supernatant at a final concentration of 0.2 mM, and the reaction mixture was incubated on ice for 10 min. Then, a final concentration of 50 mM Tris–HCl (pH 8.0) buffer was added to the reaction mixture at room temperature for 30 min to quench the reaction. The results were analyzed by immunoblotting.

### Size exclusion chromatography

The size exclusion chromatography assay was conducted using a Superdex 200 Increase 10/300 GL column (GE Healthcare) on a GE ÄKTA pure machine. The cells were collected and lysed by sonication. 500 μL of supernatant was loaded onto the column and fractionated at a flow rate of 0.5 mL/min. The elution fractions were analyzed by immunoblotting.

### Immunoprecipitation

Briefly, plasmids were transfected into HEK293T cells using polyethylenimine. 48 h after transfection, cells were collected and resuspended in lysis buffer containing 50 mM Tris–HCl (pH 7.5), 300 mM NaCl, and 1.0% (*v*/*v*) Triton X-100 supplemented with 1 mM PMSF and complete protease inhibitor cocktail. The cell lysates were immunoprecipitated with anti-HA or Ni IDA Beads at 4 °C overnight. Then, the beads were washed three times with lysis buffer at 4 °C, and the immunoprecipitated samples were analyzed by immunoblotting.

### NADK enzymatic assay

The NADK enzymatic assay was performed as described previously.^24^ Briefly, 0.5 μg of purified NADK or NADK variant proteins were used for NADK enzymatic assay. Each reaction system contained 100 μL of reaction buffer: 10 mM glucose-6-phosphate, 0.5 U G6PD, 10 mM ATP, 10 mM MgCl_2_, 100 mM Tris–HCl (pH 8.0), and varying concentrations of the substrate NAD^+^. The change in optical density at 340 nm (OD340) was measured at 37 °C every 2 min for 20 min. Experimental data were analyzed by GraphPad Prism 7 and OriginPro 9.1.

### Isothermal titration calorimetry (ITC) assay

The ITC assay was conducted using a MicroCal PEAQ-ITC instrument. Following cleaning of the sample cell and syringe, purified NADK or NADK variant proteins were carefully injected into the sample cell using a bubble-free micro-syringe. The substrate molecule NAD^+^ was introduced into a titration syringe, while deionized water served as a heat balance control in the reference cell. Both protein and NAD^+^ were prepared in 50 mM Tris–HCl buffer (pH 7.5) and 500 mM NaCl. After 19 titrations of the sample cell with NAD^+^ at a constant rate of 150 s, the titration curve was fitted using a nonlinear least squares estimation model in the MicroCal PEAQ-ITC Analysis Software (version 1.41).

### EdU incorporation assay

The EdU incorporation assay was performed using an EdU Cell Proliferation Image Kit (Abbkine, #KTA2030) according to the manufacturer's instructions. Briefly, cells were seeded on coverslips and cultured overnight before exposure to a medium containing 10 μM EdU for 2 h. Following fixation and permeabilization, the cells were treated with the Click-iT reaction mixture at room temperature for 30 min in the dark. The cells were washed three times with PBS and stained with DAPI (Biolegend, #422801), and then the coverslips were mounted on glass slides. Images were captured by Nikon eclipse-Ti2 fluorescence microscopy, and EdU-positive cells were quantified and analyzed.

### Colony formation assay

Cells were inoculated in 3.5 cm culture dishes at a density of 800 cells per well. After 8–10 days of culture, colonies were washed with PBS three times, fixed with 4% paraformaldehyde for 15 min, and then stained with 0.1% crystal violet for 2 h. After the residual crystal violet was washed away with PBS, the total area of colonies was measured by ImageJ 1.52a Software.

### Measurements of NADP^+^ and NADPH

NADP^+^ and NADPH levels were measured using a NADP^+^/NADPH colorimetric quantification assay (Sigma, #MAK038) as described previously.^24^ Cells were collected and lysed in pre-cooled 80% methanol and then centrifuged at 16,000 *g* for 10 min. The supernatant was dried down under nitrogen gas. Metabolites were then resuspended in 200 μL of extraction buffer and divided into two halves. One half was incubated at 60 °C for 30 min to decompose NADP^+^, and the other half was left on ice for 30 min. 50 μL of the samples were added to 96-well plates followed by the addition of 100 μL of master reaction mix. The plates were incubated at room temperature for 10 min and then the NADPH developer was added. The optical density at 450 nm (OD450) was measured at 15-min intervals over 2 h. Finally, 10 μL of stop solution was added to each well to terminate the reaction.

### Measurement of intracellular ROS

Intracellular ROS levels were assessed using the ROS assay kit (Dojindo, #R253) following the manufacturer's instructions. Cells were incubated with the ROS assay working solution at 37 °C for 30 min. After washed twice with PBS, the cells were collected after digestion with trypsin. The ROS levels were measured by flow cytometry using Beckman CytoFLEX.

### Cell growth assay

Cells were seeded in 96-well plates at a density of 1000 cells per well. After the cells adhered to the plate, the initial cell numbers were measured according to the manufacturer's instructions. Briefly, the Cell Counting Kit 8 (CCK8) reagents (Dojindo, #CK04) were added to each well and the plates were incubated at 37 °C for 2 h. Then, the optical density at 450 nm (OD450) was measured using a plate reader. The cell numbers were measured again by reading OD450 values at 24, 48, and 72 h after initial measurement. The cell proliferation rates were analyzed using GraphPad Prism 7.0.

### Drug response experiment

Cells were seeded in 96-well plates at a density of 1000 cells per well. After adherence, the cells were treated with serial concentrations of cisplatin (MCE, #HY-17394) for 4 days. Cell numbers were measured using the CCK8 reagents by reading OD450 values. Finally, the IC50 values of the drug were determined by nonlinear regression using GraphPad Prism 7.0.

### Xenograft tumor growth assay

In the tumor growth assay, 1 × 10^6^ H520 cells were subcutaneously injected into the armpits of nude mice. Once tumors developed, the mice were divided into two groups: a control group and a cisplatin-treatment group. Mice in the control group received intraperitoneal injections of 200 μL PBS every 3 days, while mice in the cisplatin-treatment group were injected with cisplatin (MCE, #HY-17394) at a dosage of 2 mg/kg every 3 days for 2 weeks. Tumor dimensions, including length and width, were measured every 3 days. Tumor volume was calculated using the formula (length × width × width)/2. Xenografts were harvested and weighed after an additional 7 days of growth.

### Sequence alignments

The amino acid sequences of NADK from various species were retrieved from Uniprot (http://www.uniprot.org/). Sequence alignment was performed using Clustal X2 and visualized in Jalview 2.11.

### Human NADK mutation data

The mutation sites and frequencies of NADK across different tissues were sourced from the COSMIC website (https://cancer.sanger.ac.uk/cosmic). The data were analyzed in GraphPad Prism 7.0 software.

## Results

### Cryo-EM structure of human NADK tetramer

**Figure 1:**
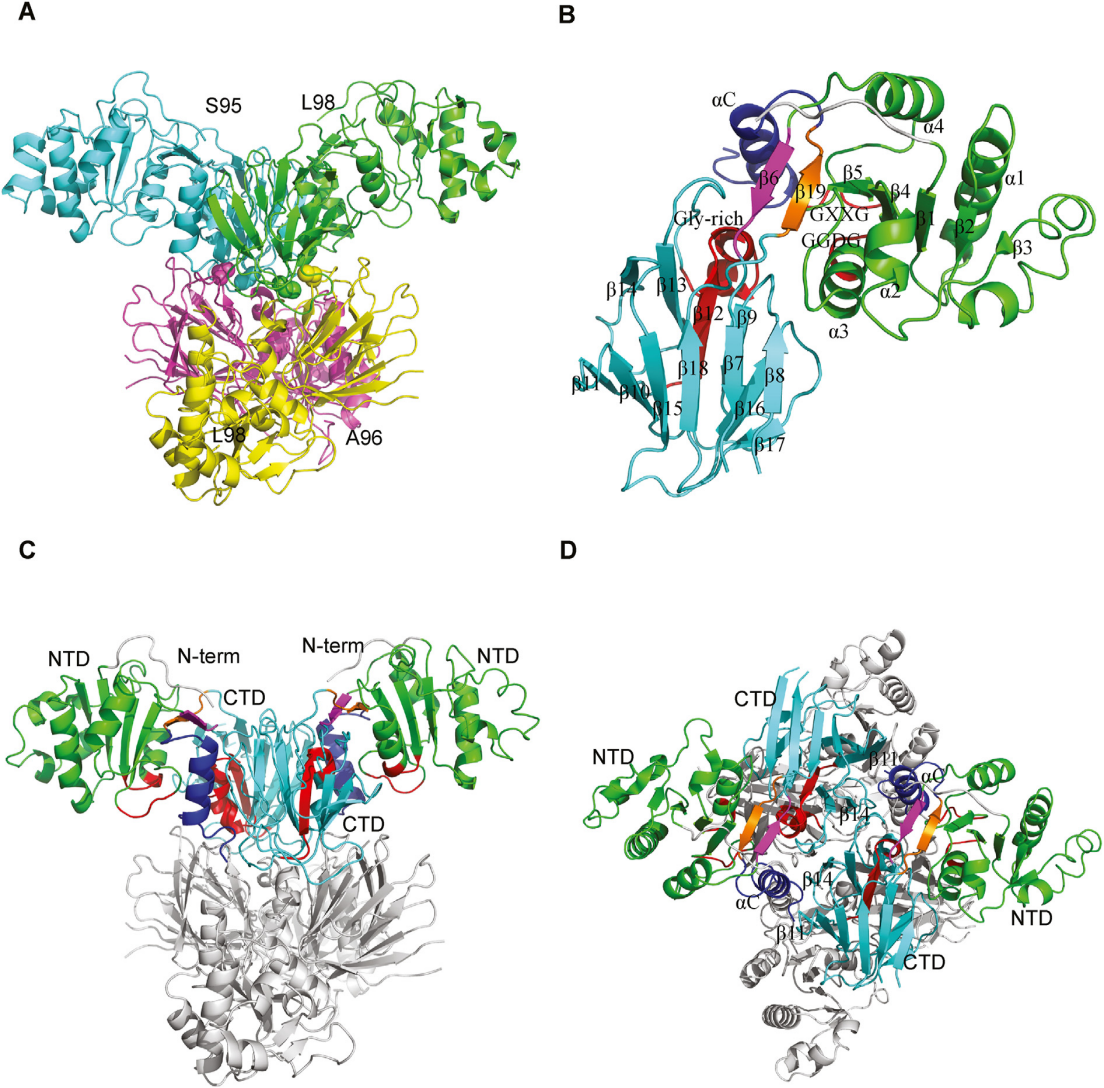
Cryo-EM structure of full-length human NADK. **(A)** Tetrameric organization of human NADK. Four protomers are presented in different colors, and the N-terminal residues of each protomer are labeled. **(B)** Close-up view of NADK monomer. The N-terminal region (grey), N-terminal domain (NTD; green), C-terminal domain (CTD; cyan), connecting β strands 6 and 19 (magenta and orange), C-terminal α helix (blue), and three glycine-rich motifs (red) in a NADK monomer are color-coded, and secondary structural modules are labeled. **(C, D)** Two different views of NADK subdomains in the tetramer.

To ascertain the detailed structural basis of enzymatic catalysis and the regulatory mechanisms of NADK, full-length NADK protein was purified for cryo-EM single-particle studies. The tetrameric structure of human NADK was determined at 2.54 Å resolution (Fig. S1), revealing a dimer of dimers arrangement. We are particularly interested in the N-terminal region because human NADK contains an additional N-terminal sequence (1–104) to the conserved catalytic domain compared with prokaryotic NADK (Fig. S2),^22^ and a few post-translational modifications have been identified in this region. However, no density was identified for this fragment in our structure; the N-terminal residues of the four protomers extend to 98, 95, 96, and 98, respectively (Fig. 1A), indicating the dynamic nature of this regulatory domain.

The NADK catalytic domain can be divided into an N-terminal domain (NTD, 105–233) and a C-terminal domain (CTD, 238–402). The NTD is a classical α/β dinucleotide binding domain, containing 5 parallel β strands (β1∼β5) flanked by 4 helices (α1∼α4) on both sides. The CTD is a β sandwich consisting of 12 anti-parallel β strands (β7∼β18) and a short helix (α5) between strands β12 and β13. The two domains are connected by a pair of anti-parallel β strands formed by a domain-linker strand (β6, 234–237) and a C-terminal strand region (β19, 405–408). These two anti-parallel β strands are located between NTD and CTD, covered by the N-terminal regulatory region, and followed by a C-terminal α helix (αC) (Fig. 1B). Sequence alignment identified three conserved motifs in the catalytic domain: a GGDG motif (182–185), GXXG motif (207–210), and a Gly-rich motif (313–332) (Fig. S2), which are located at the NTD/CTD interface and are critical for substrate binding and catalysis (Fig. 1B). Although no substrate bound in this cleft in the structure, it is reasonable to predict from structural alignment that Tyr295 and Tyr327 should be critical for NAD^+^/NADP^+^ binding; they contribute to the π-π stacking interaction with the pyridine ring of nicotinamide when NAD^+^ or NADP^+^ bind here, or interact with the adenosine ring when ATP binds. Additionally, Asn284 and Glu285 can interact with the pentose sugar in the substrate. Asn284, Glu285, and Tyr327 are conserved (Fig. S2). The invariant Asn184 in the conserved GGDG motif is close to the 2′ sugar-phosphate group of NADP^+^, suggesting a catalytic role in phosphorylating NAD^+^.^33^

Strands β11 and β14 in the CTD domain mediate the dimerization, along with the C-terminal αC helix from the neighboring monomer (Fig. 1C, D). The αC helix is situated at the dimer interface, establishing extensive contact with both the NTD and the neighboring CTD. Subsequently, the CTD further mediates the tetramerization (Fig. 1C, D). Given its proximity to the substrate binding site, the αC helix may play a regulatory role in NADK activity.

### Regulating NADK tetramer conformation by the N-terminal region

**Figure 2:**
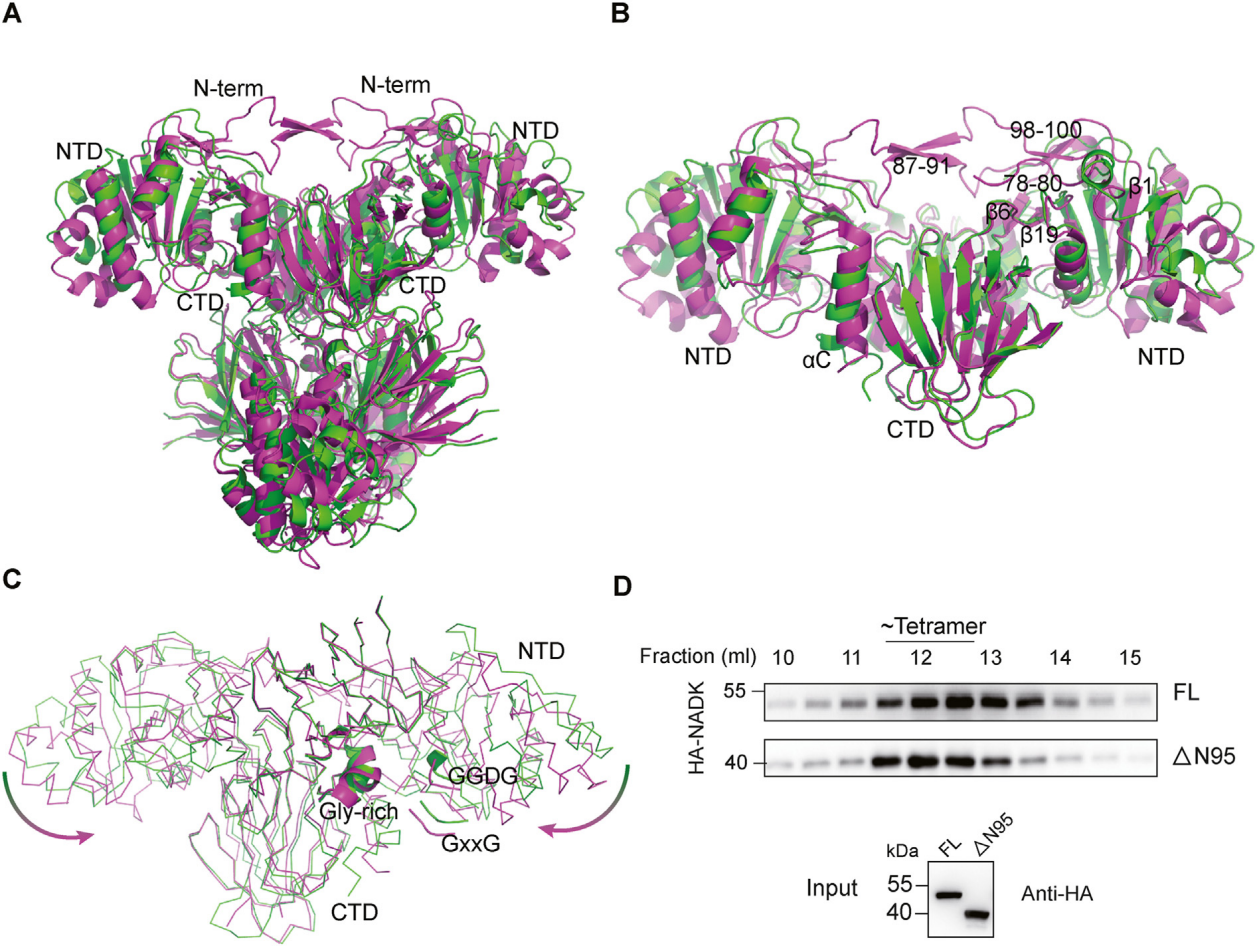
N-terminal region regulates NADK conformation. **(A)** Comparison of the cryo-EM structure (green, pdb: 8KGC) and the crystal structure (purple, pdb: 3PFN) of tetrameric NADK. **(B)** Close-up view of the NADK dimer involving the N-terminal regulation. Two pairs of anti-parallel β strands, the first β1 strand in the N-terminal domain (NTD), the linker anti-parallel β strands (β6 and β19), and the C-terminal αC helix are labeled. **(C)** NADK dimer presented in ribbon, highlighting the GGDG, GxxG, and the Gly-rich motifs. The arrows indicate the rotation of NTD around the linker anti-parallel β strands. **(D)** The indicated NADK full-length (FL) and N-terminal truncation mutant (ΔN95) plasmids were introduced into HEK293T cells with endogenous NADK knocked out. HA-NADK was eluted in size exclusion fractions on the same column in identical settings. Indicated fractions of cell lysates were used for immunoblot analyses.

The structure of human NADK tetramer was previously determined by X-ray crystallography (pdb code: 3PFN). Both the crystal structure and the cryo-EM structure are substrate-free tetramers. While the overall tetrameric organization is similar, significant differences are observed in local regions, especially in the relative orientation of the NTD and CTD domains (Fig. 2A). The r.m.s.d. values of the NTD and CTD between the crystal and cryo-EM structures are 0.99 and 0.42 Å, respectively (Fig. S3), indicating that the CTD is more rigid than the NTD domain. Flexibility in the NTD region is mainly localized to the region before the β1 strand and the linker region connecting the β2 to β4 strands (Fig. S3A), while the CTD is generally rigid, with only the C-terminal αC helix being flexible (Fig. S3B). The comparison of the two structures demonstrates that the tetrameric core, formed by the 4 CTDs, is relatively stable. The major difference is caused by the interaction of the N-terminal region within the dimers, which regulates the orientation of the NTD and further affects substrate binding and NADK activity. Although we were interested in the regions containing the post-translational modification sites (including phosphorylation (Ser44, Ser46, and Ser48)^24^ and methylation (Arg39, Arg 41, and Arg 45)), they are not visible in both structures.

In the crystal structure 3PFN, the N-terminal extends to residues 72, 69, 72, and 69, respectively, generally longer than that in the cryo-EM structure. Interestingly, a N-terminal region (residues 87–91) from two protomers forms anti-parallel β strands that interact with each other in the dimer (Fig. 2B). This interaction pushes the following region towards the NTD, resulting in the formation of additional anti-parallel β strands (strand 98–100 and strand 78–80 from the neighboring molecule) just before the first β1 strand in the NTD (Fig. 2B). Thus, the mechanical force from the N-terminal region pushes the NTD to rotate around the linker anti-parallel β strands (β6 and β19), resulting in the GGDG and GXXG motifs to move towards the Gly-rich motif (Fig. 2C). All these findings support the role of the N-terminal region in regulating NADK tetramer conformation, which may affect its activity. Although the N-terminal region is disordered in our cryo-EM structure, NADK still forms a tetramer, indicating that the N-terminal region is not necessary for tetramer formation. Supporting this concept, the N-terminal truncated NADK (95–446) still forms a tetramer (Fig. 2D). The tetrameric structure of NADK, following the truncation of its N-terminus, appears to exhibit enhanced homogeneity. This enhanced homogeneity may be attributed to the absence of regulatory control exerted by the N-terminal region on the overall structure.

Our cryo-EM structure represents a status in solution, in which the N-terminal region is more flexible, and no anti-parallel β strands are formed in this region. Thus, the N-terminal is disordered, and the strands 78–80 from the neighboring molecule are not in place, preventing the region 98–100 forming anti-parallel β strands with strands 78–80 (Fig. 2B). As a result, there is no mechanical force to push the NTD to rotate towards CTD, causing the NTD to extend outward compared with 3PFN, leading to a looser tetramer. The rare isoform 3 of NADK, which lacks the N-terminal regulatory domain 24, may adopt a similar conformation. The highly active isoform 3 suggests that the looser NADK tetramer is better suited for substrate binding and release, leading to higher activity.

### Requirement of NADK tetramer for its enzyme activity

**Figure 3:**
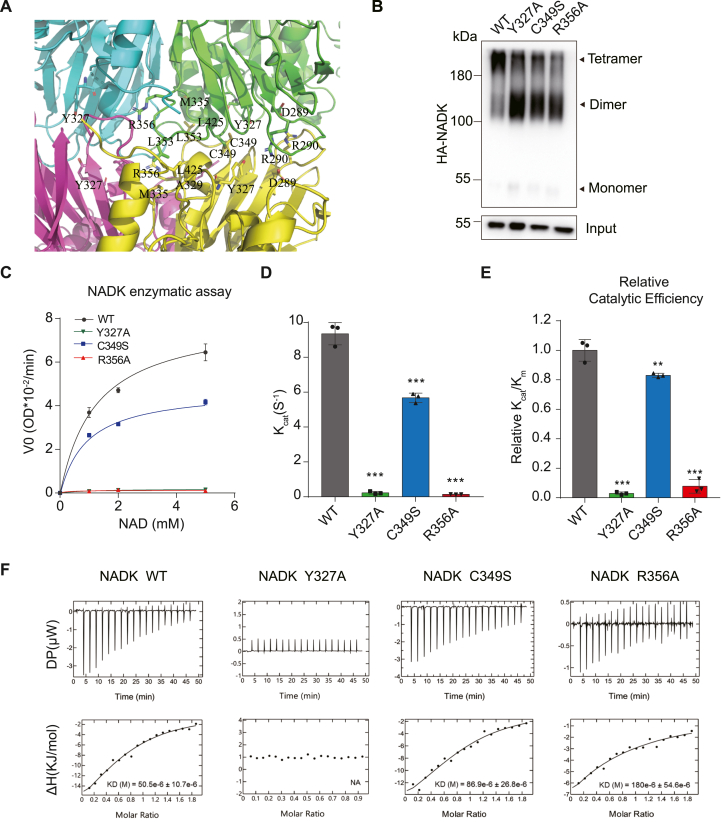
NADK tetramer is required for its enzyme activity. **(A)** Tetramer interface of NADK. C349 and R356 on the interface, and the hydrophobic interaction and salt bridges are highlighted and labeled. **(B)** NADK WT and indicated mutant plasmids were transfected into HEK293T cells. The cell lysates were cross-linked with 0.2 mM BS3 and terminated with 50 mM Tris–HCl (pH 8.0). NADK input and cross-linked fractions were detected by immunoblot analyses. **(C**–**E)** NADK enzymatic assay with purified NADK WT and indicated mutant proteins from *E. co*li BL21 strain (*n* = 3). Michaelis–Menten curve (C), K_cat_ (D), and relative catalytic efficiency (E) are presented. **(F)** ITC binding curves for NADK WT and indicated mutant proteins with substrate molecule NAD^+^. The Kd values, when measurable, are shown in the figure, while non-measurable values are indicated by NA. Data in (C–E) are presented as mean ± standard deviation; one-way ANOVA; ∗*p* < 0.05, ∗∗*p* < 0.01, and ∗∗∗*p* < 0.001.

The above analysis reveals a conformational change in NADK which regulates the substrate binding site, with the N-terminal region playing a regulatory role in this process. This conformational change occurs within the NADK dimer, which further assembles into a tetramer (a dimer of dimers). The tetramer interface is formed by 3 loop regions of the CTD β strands, including hydrophobic interactions (Leu353 contacts with Ala329, Met335, and Leu425 from neighboring molecule) and salt bridges (Asp289 and Arg290 form two pairs of salt bridges with the neighboring molecule) (Fig. 3A). Notably, Cys349 is located at the tetramer interface, positioned near another Cys349 from the adjacent dimer, enabling the formation of an intermolecular disulfide bond (Fig. 3A). Thus, in addition to the non-covalent interactions, covalent disulfide bonds also contribute to the stabilization of the tetramer. To investigate the involvement of other cysteine residues in the formation of disulfide bonds, cysteines located within the N-terminal region and those identified as potential participants based on structural analysis were screened using a gel-filtration assay. The results revealed that solely the mutation at the C349 site impacted the NADK tetramer assembly (Fig. S4A). Consistently, a cross-linking experiment showed that much less tetramer was detectable in a C349S mutant compared with NADK WT (Fig. 3B). To understand if the NADK tetramer affected its activity, purified WT and C349S proteins were used for NADK activity assay. The results showed that the C349S mutant had a reduced activity (Fig. 3C–E), indicating that a stable NADK tetramer facilitates its enzyme activity.

Next, we generated two mutations on the tetramer interface. Arg356 is on the tetramer interface which makes contacts with the main chain of the two neighboring molecules (Fig. 3A). Tyr327 is located at the tetramer interface, mediating tetramer formation, with the phenyl group of Tyr forming π-π interactions with the pyridine ring of nicotinamide, making it a critical residue for substrate binding. When these two residues were mutated to Ala, as expected, both mutations diminished tetramer organization (Fig. 3B), as observed with C349S did in the cross-linking experiment. While C349S reduced NADK activity, mutation of these residues resulted in a total loss of NADK activity (Fig. 3C–E). These results indicated that NADK tetramer was required for its activity.

### Tetramer enhances substrate binding

To understand how the NADK tetramer affected its activity, we conducted ITC assays using purified proteins to measure substrate binding affinity. As depicted in Figure 3F, the C349S mutant slightly reduced the affinity to NAD^+^ compared with the WT, indicating that a stable tetrameric conformation is conducive to substrate binding. From a structural view, the Y327A mutant directly affects NAD^+^ binding, it indeed completely abolishes it (Fig. 3F). Notably, the R356A mutant, which disrupts tetramer conformation but has no direct contact with the substrate-binding site, also significantly affected NAD^+^ binding, with the dissociation constant value (Kd) increasing from 50.5 mM to 180 mM (Fig. 3F). Taken together, these results suggested that stable tetramer formation enhances the substrate NAD^+^ binding, and mutations affecting tetramer formation may affect its activity.

As illustrated in Figure S4B, the three mutants related to NADK tetramer formation are highly conserved. To investigate whether these NADK mutations exhibited similar biological effects *in vivo*, we complemented these mutants in NADK knockout lung cancer H520 cells (Fig. S4C). We found that these NADK mutants attenuated the rate of EdU incorporation and cell proliferation (Fig. S4D, E). Consistently, these mutants were unable to restore the production of NADP^+^ and NADPH compared with the WT (Fig. S4F, G). These results further supported that tetramer formation affects the biological function of NADK and cell growth.

### Methylation site mutation increases NADK activity

**Figure 4:**
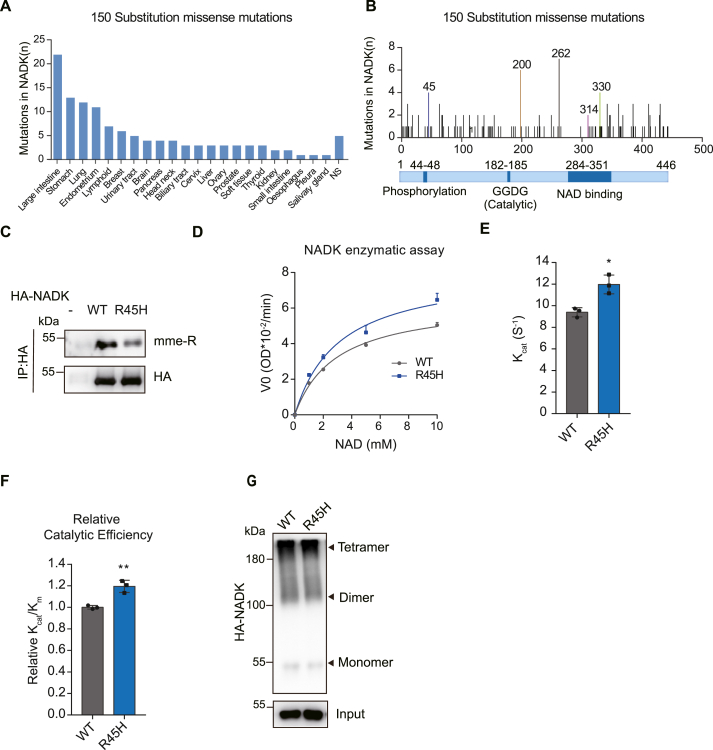
R45H mutation increases NADK activity. **(A)** The mutation frequency (mutated cases/total cases) of NADK across 22 human tissue types from the COSMIC database. **(B)** The distribution of substitution missense mutation positions of NADK from the COSMIC database. **(C)** The methylation levels of NADK WT and R45H mutant proteins were quantified by immunoblotting with anti-MME-R antibody. **(D**–**F)** NADK enzymatic assay with purified NADK WT and R45H mutant proteins from HEK 293T cells (*n* = 3). Michaelis–Menten curve (D), K_cat_ (E), and relative catalytic efficiency (F) are presented. **(G)** NADK WT and R45H mutant plasmids were transfected into HEK293T cells. The cell lysates were cross-linked with BS3. NADK input and cross-linked protein samples were detected by immunoblot analyses. Data in (D–F) are presented as mean ± standard deviation; one-way ANOVA; ∗*p* < 0.05, ∗∗*p* < 0.01, and ∗∗∗*p* < 0.001.

Analysis from the Catalogue of Somatic Mutations in Cancer (COSMIC) database revealed that 580 NADK mutations were detected in 46,086 samples, with 26% being substitution missense mutations. These mutations were mainly clustered in intestinal cancer, gastric cancer, and lung cancer (Fig. 4A). Notably, hot spots with the highest mutation frequency include R45, P200, N262, and A330 (Fig. 4B). A frequently occurring mutation in the N-terminal region, R45H (highly frequent mutation in lung cancer), was identified. R45 is located within the phosphorylation region, which is highly conserved (Fig. S5A). Additionally, arginine methylation by PRMT6 has been identified on this site to inhibit NADK activity.^34^ Pursuing the N-terminal regulatory role of NADK further, we examined the R45H mutation. We found that the R45H mutant weakened the NADK arginine mono-methylation (Fig. 4C). An *in vitro* NADK enzymatic assay using NADK protein expressed and purified from 293T cells showed that the R45H mutant strengthened NADK enzyme activity (Fig. 4D–F). However, when the recombinant protein was expressed and purified from the *E. coli* BL21 strain for the activity assay, no difference was observed in activity (Fig. S5B–D). This difference could be attributed to the lack of relevant modifications at the R45 site in prokaryotic cells (Fig. S5E). These results indicated that the methylation on NADK R45 attenuates NADK activity. We next investigated how R45H affected NADK tetramer or substrate binding. Consistent with the N-terminal deletion, the R45H mutant did not affect tetramer formation (Fig. 4G). ITC experiments showed that the R45H mutation slightly affected the binding of its substrate NAD^+^ molecule to NADK (Fig. S5F).

### Methylation site mutation promotes tumor growth and confers chemotherapy resistance

**Figure 5:**
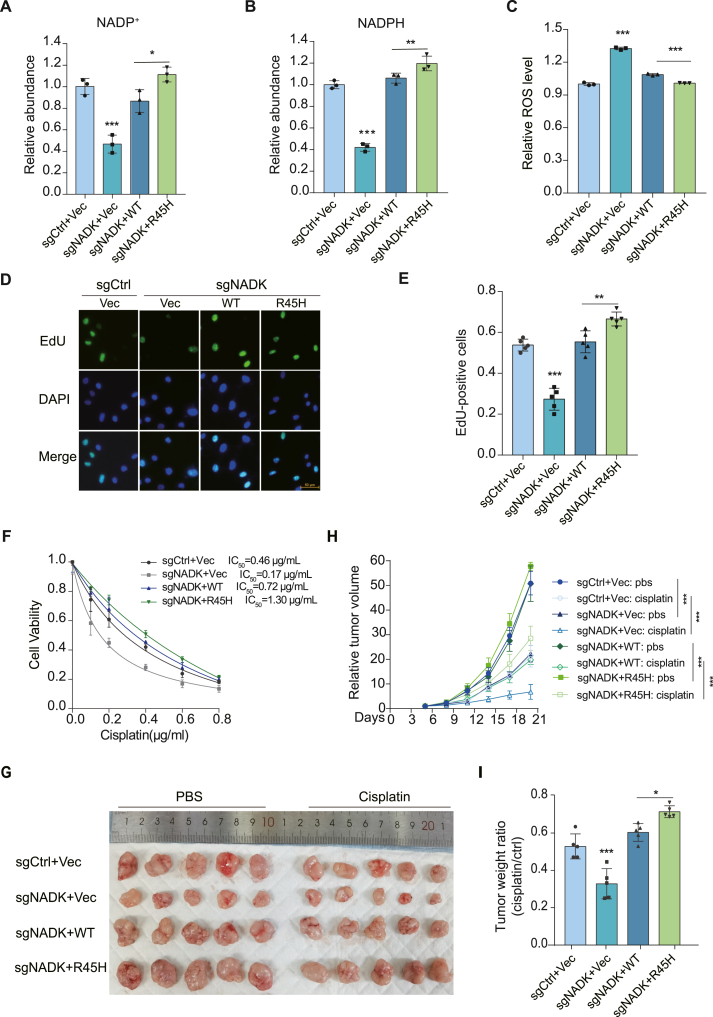
R45H mutation promotes tumor growth by reducing reactive oxygen species (ROS) levels. **(A, B)** H520 cells with stable sgRNA-mediated knockout of NADK were stably reconstituted with NADK WT or R45H mutant. The relative abundance of NADP^+^ (A) and NADPH (B) quantified from these reconstituted H520 cell lines was presented (*n* = 3). **(C)** The reconstituted H520 cells were incubated with the ROS assay working solution at 37 °C for 30 min. Relative ROS level was quantified by flow cytometry (*n* = 3). **(D, E)** The reconstituted H520 cells were labeled with 10 μM EdU for 30 min, and EdU-positive cells were examined by immunostaining test (D). EdU-positive cells are presented in (E) (*n* = 5). Scale bars: 50 μm. **(F)** The reconstituted H520 cells were exposed to indicated concentrations of cisplatin for 96 h, and cell viability assay was performed (*n* = 4). The IC_50_ of cisplatin was calculated. **(G**–**I)** Mice were subcutaneously injected with 1 × 10^6^ of the indicated H520 cells on both sides of the armpit (*n* = 5). When tumors were visible, mice were treated with cisplatin (2 mg/kg) every 3 days for 2 weeks. The tumors were harvested after an additional 7 days of growth (G). Relative tumor volume (H) and tumor weight ratio (cisplatin/ctrl) (I) are presented. Data in (A–F, I) are presented as mean ± standard deviation; one-way ANOVA; ∗*p* < 0.05, ∗∗*p* < 0.01, and ∗∗∗*p* < 0.001. Data in (H) are presented as mean ± standard deviation; two-way ANOVA; ∗*p* < 0.05, ∗∗*p* < 0.01, and ∗∗∗*p* < 0.001.

To study the *in vivo* function of R45H mutation, NADK knockout H520 cells were reconstituted with NADK WT or R45H mutant (Fig. S5G). The NADK R45H mutant generated more NADP^+^ and NADPH compared with that of WT cells (Fig. 5A, B), conforming to the higher activity of the R45H mutation. Moreover, we detected the ROS levels in these cells. NADK knockout H520 cells exhibited a significantly increased intracellular ROS level compared with the parental cells. Reconstitution of NADK WT rescued the ROS level, and the R45H mutation decreased the ROS level compared with the WT (Fig. 5C), presumably due to the increase in NADPH level caused by the higher NADK activity. Additionally, the EdU incorporation assay showed that NADK knockout H520 cells had a reduced cell proliferation rate compared with the parental cells; this was restored by the introduction of NADK WT, and the R45H mutation further increased cell proliferation (Fig. 5D, E). Consistently, similar results were obtained from cell proliferation and colony formation assays (Fig. S5H–J).

We further explored the impact of NADK activity on the response of tumor cells to chemotherapy drugs. NADK knockout H520 cells exhibited the smallest IC50 (0.17 μg/mL) for cisplatin, indicating increased sensitivity to cisplatin and highlighting the important role of NADK in drug sensitivity. Tumor cells with the R45H mutant had a higher IC50 (1.30 μg/mL) than those with NADK WT (0.72 μg/mL), suggesting that higher NADK activity attenuates the chemotherapy response (Fig. 5F). To validate these findings *in vivo*, we conducted animal studies by implanting these modified H520 cells into BALB/c nude mice and then monitored tumor growth and chemotherapy response. The deficiency of NADK greatly inhibited tumor growth, and the reconstruction of NADK WT restored the tumor growth, whereas the NADK R45H mutant resulted in a larger tumor compared with NADK WT (Fig. 5G, H). Furthermore, the NADK knockout tumors were more sensitive to cisplatin. Contrarily, NADK R45H mutant tumors were more resistant to cisplatin than NADK WT tumors (Fig. 5I), indicating that the up-regulated NADK activity confers resistance to chemotherapy in lung cancer cells.

### Cancer-related NADK mutants modulate the tetramer conformation and NADK activity

**Figure 6:**
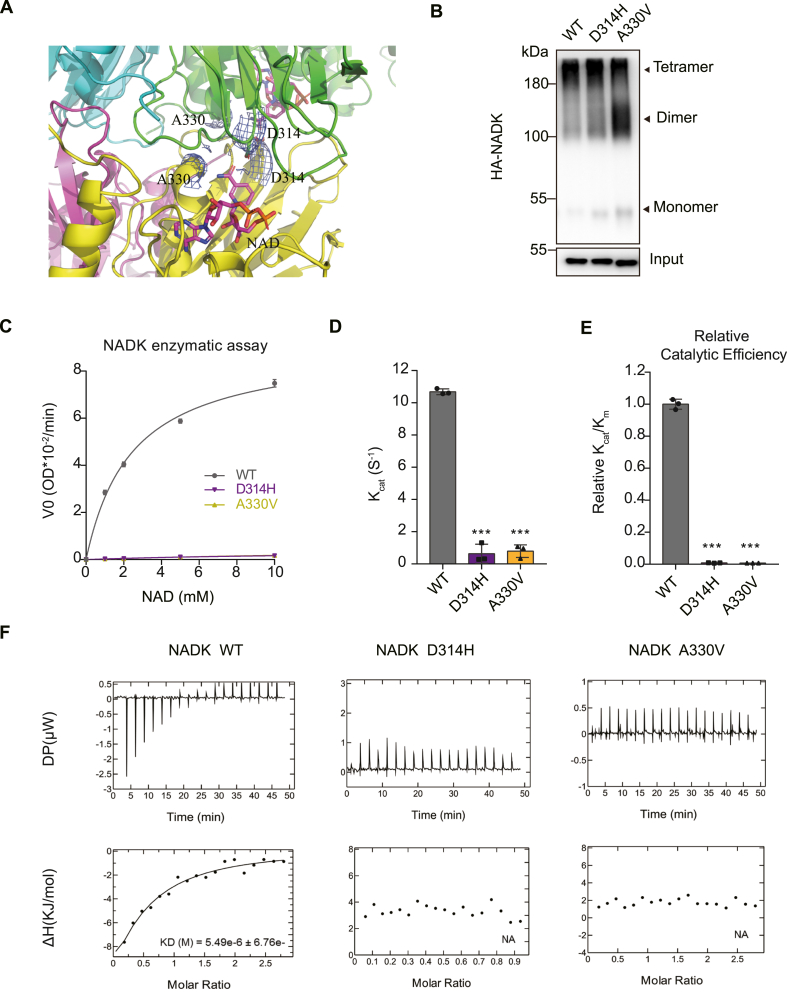
Tetramer interface mutations modulate NADK activity. **(A)** The electronic density of D314 and A330 is presented on the NADK tetramer. **(B)** NADK WT and indicated mutant plasmids were transfected into HEK293T cells. The cell lysates were cross-linked with BS3. NADK input and cross-linked fractions were detected by immunoblot analyses. **(C**–**E)** NADK enzymatic assay with purified NADK WT and indicated mutant proteins from *E. coli* BL21 strain (*n* = 3). Michaelis–Menten curve (C), K_cat_ (D), and Relative catalytic efficiency (E) are presented. **(F)** ITC binding curves for NADK WT and indicated mutant proteins with substrate molecule NAD^+^. The Kd values, when measurable, are shown in the figure, while non-measurable values are indicated by NA. Data in (C–E) are presented as mean ± standard deviation; one-way ANOVA; ∗*p* < 0.05, ∗∗*p* < 0.01, and ∗∗∗*p* < 0.001.

Given the significance of the NADK tetramer for its activity, it is reasonable to hypothesize that mutations decreasing the NADK tetramer may diminish NADK function. To comprehend the relationship between the NADK tetramer and cancer development and treatment, we investigated mutants located on the tetramer interface that frequently occur in human cancers. Ala330 is located at the tetramer interface (Fig. 6A), and it also interacts with the substrate NAD^+^ (nicotinamide group), potentially making it critical for NADK activity; it is frequently altered to Thr or Val in cancers from the COSMIC database (Fig. 4A and B). Indeed, the A330V mutation disrupts the tetramer structure (Fig. 6B) and leads to a reduction in NADK activity (Fig. 6C–E). ITC experiments demonstrate a significant decrease in the affinity of the A330V mutant to NAD^+^ (Fig. 6F). Similarly, Asp314, located at the side of the tetramer interface (Fig. 6A), is frequently mutated to His (Fig. 4A, B). While the D314H mutation does not significantly impact tetramer formation (Fig. 6B), it abolishes NADK activity (Fig. 6C–E) by affecting the binding affinity to NAD^+^ (Fig. 6F).

### Loss-of-function NADK mutations from cancer patients enhance the sensitivity of chemotherapy

**Figure 7:**
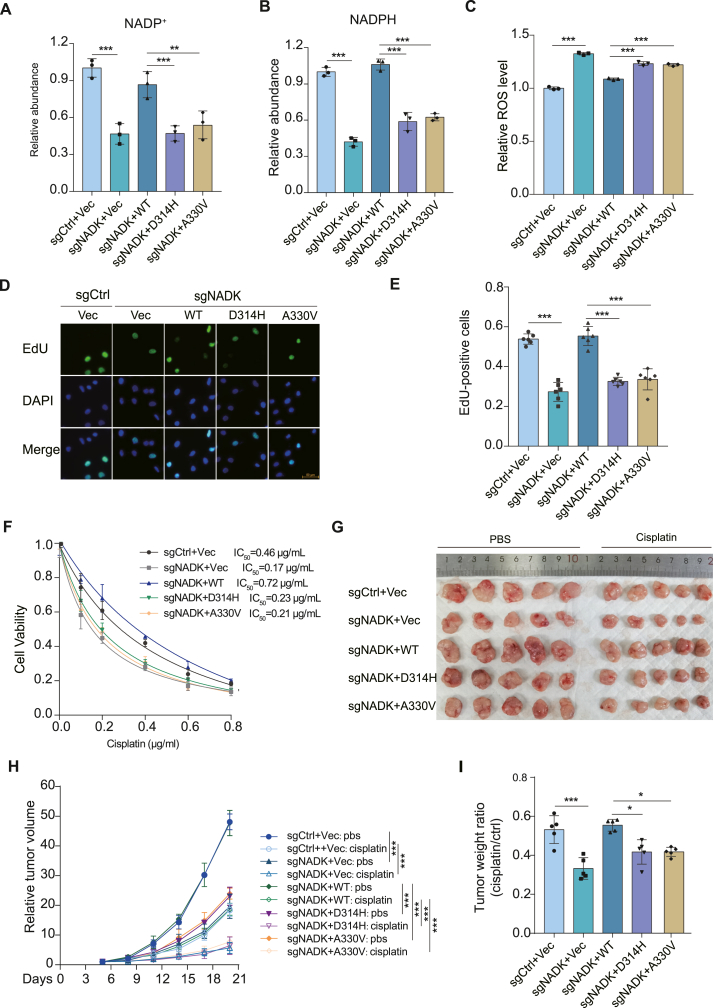
Tetramer interface mutations affect cancer cell proliferation and increase chemotherapy response. **(A, B)** Relative abundance of NADP^+^ (A) and NADPH (B) quantified from these reconstituted H520 cell lines (*n* = 3). **(C)** Relative reactive oxygen species (ROS) level quantified by flow cytometry (*n* = 3). **(D, E)** The reconstituted H520 cells were labeled with 10 μM EdU for 30 min, and EdU-positive cells were examined by immunostaining test (D). EdU-positive cells are presented in (E) (*n* = 5). Scale bars: 50 μm. **(F)** The reconstituted H520 cells were exposed to indicated concentrations of cisplatin for 96 h, and cell viability assay was performed (*n* = 4). The IC_50_ of cisplatin was calculated. **(G**–**I)** Mice were subcutaneously injected with 1 × 10^6^ of the indicated H520 cells on both sides of the armpit (*n* = 5). When tumors were visible, mice were treated with cisplatin (2 mg/kg) every 3 days for 2 weeks. **(G)** The tumors were harvested after an additional 7 days of growth. **(H, I)** Relative tumor volume (H) and tumor weight ratio (cisplatin/ctrl) (I) are presented. Data in (A–F, I) are presented as mean ± standard deviation; one-way ANOVA; ∗*p* < 0.05, ∗∗*p* < 0.01, and ∗∗∗*p* < 0.001. Data in (H) are presented as mean ± standard deviation; two-way ANOVA; ∗*p* < 0.05, ∗∗*p* < 0.01, and ∗∗∗*p* < 0.001.

Next, we explored these loss-of-function mutations *in vivo*. NADK D314 and A330 sites are highly conserved across multiple species (Fig. S6A). NADK knockout H520 cells were reconstituted with NADK WT, D314H, and A330V mutants (Fig. S6B). Consistently, NADK knockout H520 cells reduced the production of NADP^+^ and NADPH, and reconstitution of NADK WT restored NADP^+^ and NADPH production, but the introduction of loss-of-function NADK mutants did not (Fig. 7A, B). On the contrary, knockout of NADK enhanced the intracellular ROS level in H520 cells, and reconstitution of NADK D314H or A330V mutant generated a higher ROS level than reconstitution of NADK WT (Fig. 7C). All these results confirmed that loss-of-function NADK mutants reduced NADP/NADPH production and leaded to an increased ROS level. Consequently, both D314H and A330V mutants inhibited cancer cell growth (Fig. 7D and E), proliferation, and colony formation (Fig. S6C–E). These loss-of-function mutations lead to an increased sensitivity to the chemotherapy drug cisplatin, as evidenced by a substantial reduction in IC50 from 0.72 μg/mL to approximately 0.2 μg/mL (Fig. 7F). In addition, *in vivo* experiments demonstrated that D314H and A330V mutants inhibited tumor growth (Fig. 7G, H) and significantly increased sensitivity to cisplatin (Fig. 7I). Consistently, the results indicated that the loss of NADK function sensitizes lung cancer to chemotherapy.

## Discussion

As the sole cytosolic enzyme that catalyzes the synthesis of NADP^+^ from NAD^+^, NADK is strictly regulated at multiple levels in cells. Here, we determine the cryo-EM structure of the full-length human NADK tetramer at 2.54 Å resolution. In comparison to bacterial NADK which consists of the essential catalytic core NTD and CTD domains,^35^ mammalian cNADK evolves an additional N-terminal regulatory domain. While not essential for NADK tetrameric organization, it dimerizes on both sides of the tetramer, positioning itself to regulate the tetramer conformation, which is critical for NADK activity. The N-terminal deletion isoform enhances NADK activity, suggesting an auto-inhibiting conformation within the N-terminal.^24^ Several regulatory pathways involving post-translational modifications on this N-terminal region, including phosphorylation and methylation, may regulate NADK activity by modulating tetramer conformation or stability, either by diminishing or enhancing the auto-inhibiting conformation.^24^ Additionally, mutations in this region may similarly affect tetramer conformation. For instance, I90 located on the anti-parallel β-strands mediating dimerization, may enhance hydrophobic interaction by forming a π-π interaction upon mutation to Phe, tightening the dimer interface between two NTDs, thereby increasing tetramer stability and activity. However, the absence of the entire N-terminal region in both X-ray and cryo-EM structures leaves the conformation of this N-terminal region a mystery. Further studies are required to understand how post-translational modifications modulate the N-terminal conformation to regulate NADK activity. Moreover, other regulatory protein binding in this region may influence NADK tetramer conformation and activity.^36^

While all bacterial NADK and mammalian cNADK form tetramers composed of dimers, with interface 1 (IF1) mediating the tetramerization and interface 2 (IF2) mediating the formation of primary dimers, surprisingly, mammalian mitochondrial NADK (mNADK, also known as NADK2) forms dimers containing only IF1. This contrasts with cNADK, where the N-terminal region is involved in dimerization. In mNADK, IF2 is interrupted by the EMKA insertion (residues 325–365), forming a long hairpin that protrudes outward.^19^ While cNADK utilizes this IF2 surface to form tetramers and employs the N-terminal region to regulate activity, mNADK lacks these regulatory properties. Furthermore, mNADK lacks the N-terminal regulatory region found in cNADK but contains a mitochondrial localization signal at its N-terminus, indicating different origins of these NADKs, and emphasizing the absence of regulatory mechanisms in mNADK. The differences provide a basis for specifically targeting cNADK.

A plethora of mutations in cNADK have been identified across a spectrum of diseases, spanning metabolic disorders, cancers, and other pathological conditions.^23^ These mutations encompass a variety of alterations, ranging from gain-of-function mutations enhancing cNADK activity to loss-of-function mutations impairing enzyme function. However, the precise underlying mechanisms driving their pathological effects and therapeutic implications remain largely elusive. Understanding the intricate interplay between cNADK mutations and disease pathology represents a complex challenge due to its multifaceted roles in cellular metabolism, signaling pathways, and redox homeostasis. Unraveling the molecular mechanisms underlying cNADK-associated diseases holds promise for developing targeted therapeutic interventions aimed at modulating NADK activity or its downstream signaling pathways.

Our study identified several cancer-associated mutants and found that one of the NADK methylation sites is mutated in certain cancer types. Specifically, the methylation-deficient mutant R45H within the N-terminal region results in decreased NADK methylation and increased NADK activity, and confers cancer chemotherapy resistance. Targeting NADK activity, such as with a specific NADK inhibitor, could re-sensitize these patients to chemotherapy. Additionally, we identified several loss-of-function NADK mutations in cancer patients, including D314H and A330V mutants. Both D314H and A330V inhibit NADK activity, reduce NADP^+^ production, and suppress tumor growth, significantly increasing sensitivity to cisplatin. Tumor cells undergo adaptive evolution through disadvantageous mutations, generating various mutations to adapt to unique tumor microenvironments. The presence of these loss-of-function mutations may reduce tumor malignancy and extend patient survival, making them easier to detect. Based on this information, these mutants could serve as powerful biomarkers for cancer chemotherapy treatment. We provide experimental evidence to support the functional consequences of specific cNADK mutations in the context of cancer development and chemotherapy.

Taken together, our findings will have a significant impact on the dissection of regulatory and catalytic mechanisms of NADK. Given that NADK is frequently mutated and linked to multiple cancer types, our findings might also have important implications for cancer etiology and response to therapy.

## CRediT authorship contribution statement

**Mengxue Hu:** Writing – original draft, Methodology, Investigation, Data curation. **Fuxing Wang:** Data curation. **Yue Zhu:** Data curation. **Yi Yao:** Resources. **Huadong Pei:** Writing – review & editing, Validation, Supervision, Methodology, Investigation, Funding acquisition, Conceptualization. **Zheng Liu:** Writing – original draft, Investigation, Data curation. **Pingfeng Zhang:** Writing – review & editing, Writing – original draft, Validation, Supervision, Investigation, Funding acquisition, Conceptualization.

## Conflict of interests

Prof. Huadong Pei is an editorial board member for *Genes & Diseases* and was not involved in the editorial review or the decision to publish this article. All authors declare that there are no competing interests.
