# Supplementary material for: NADK tetramer defective mutants affect lung cancer response to chemotherapy via controlling NADK activity
Source: Genes Dis. 2025 Jan 7;12(4):101510. doi: 10.1016/j.gendis.2024.101510 (PMC12052686; doi:10.1016/j.gendis.2024.101510)
Supplement: Multimedia component 1 [file mmc1.docx]

**
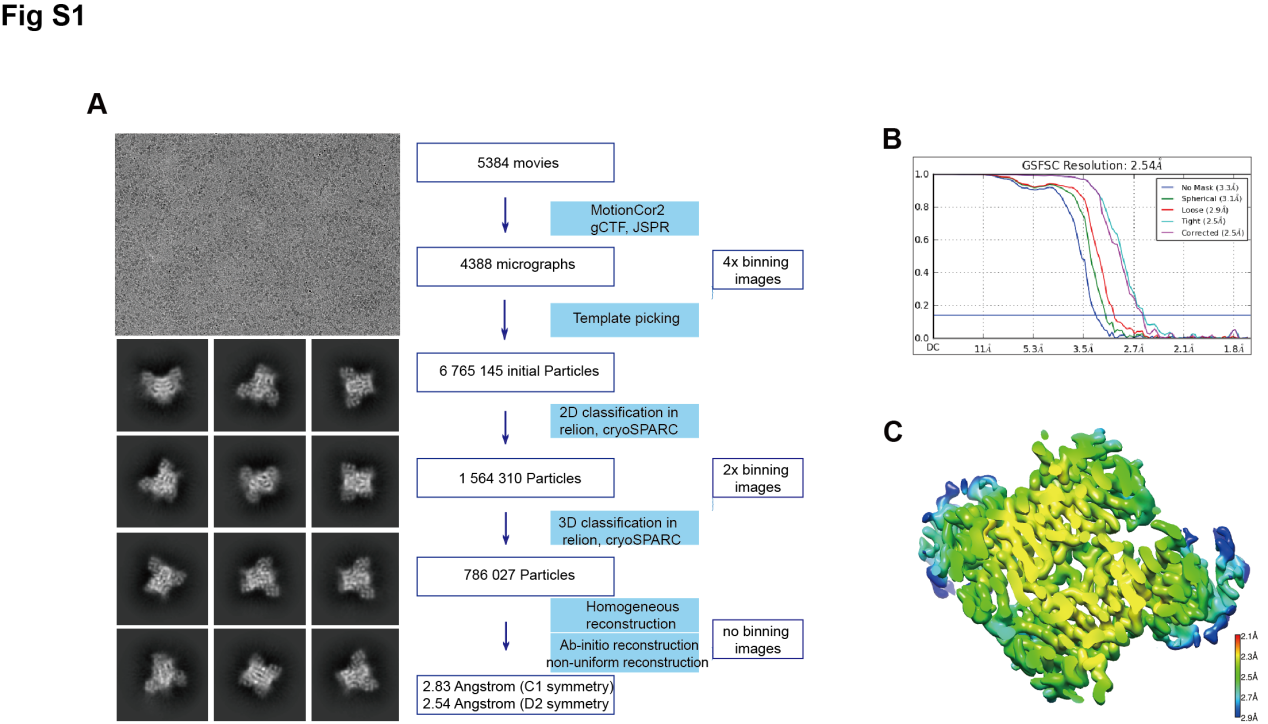
**

**Figure S1 Cryo-EM structure determination of tetrameric human NADK.**

1. Workflow of cryo-EM image processing of the NADK dataset. Top left: A representative motion-corrected cryo-electron micrograph of the NADK dataset. Bottom left: Reference-free 2D class averages, highlighting clear density for tetrameric composition. The right panel presents the preprocessing of the four-time binning images.
2. The overall resolution, as determined by a gold standard FSC (GSFSC) cut-off value of 0.143 (purple line), is 2.54 Å.
3. Local resolution distributions for the NADK structure.

**
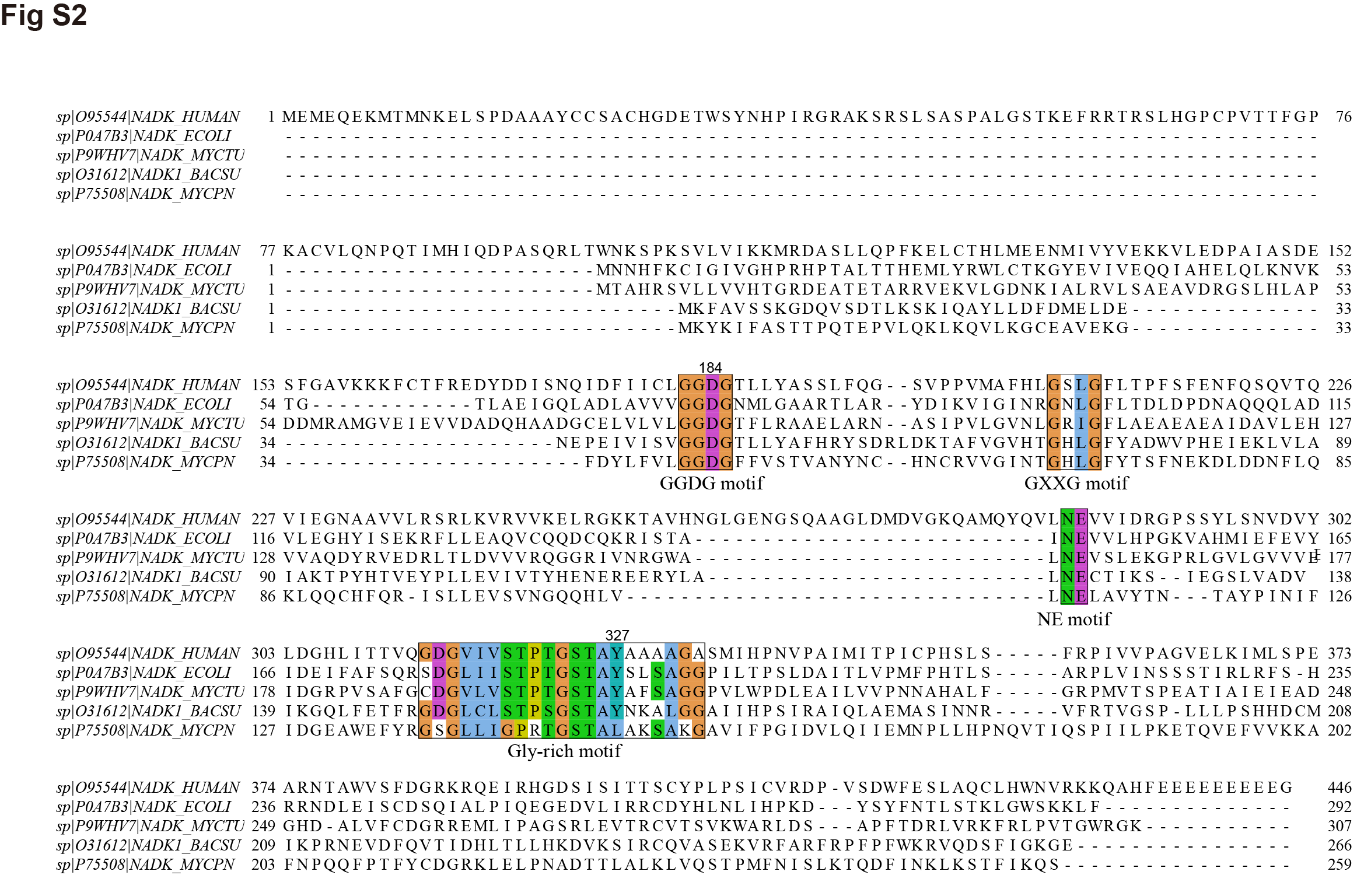
**

**Figure S2 Multiple sequence alignment of NAD+ kinases from various species.** The NADK sequences from human (*Homo sapiens*) (O95544), *Escherichia coli* (P0A47B); *Mycobacterium tuberculosis* (P9WHV7), *Bacillus subtilis* (O31612), and [*Mycoplasma pneumoniae*](https://www.uniprot.org/taxonomy/272634) ([P75508](https://www.uniprot.org/uniprotkb/P75508/entry)) were retrieved from Uniprot. Sequence alignment was performed using Clustal X2 and visualized in Jalview 2.11.

**
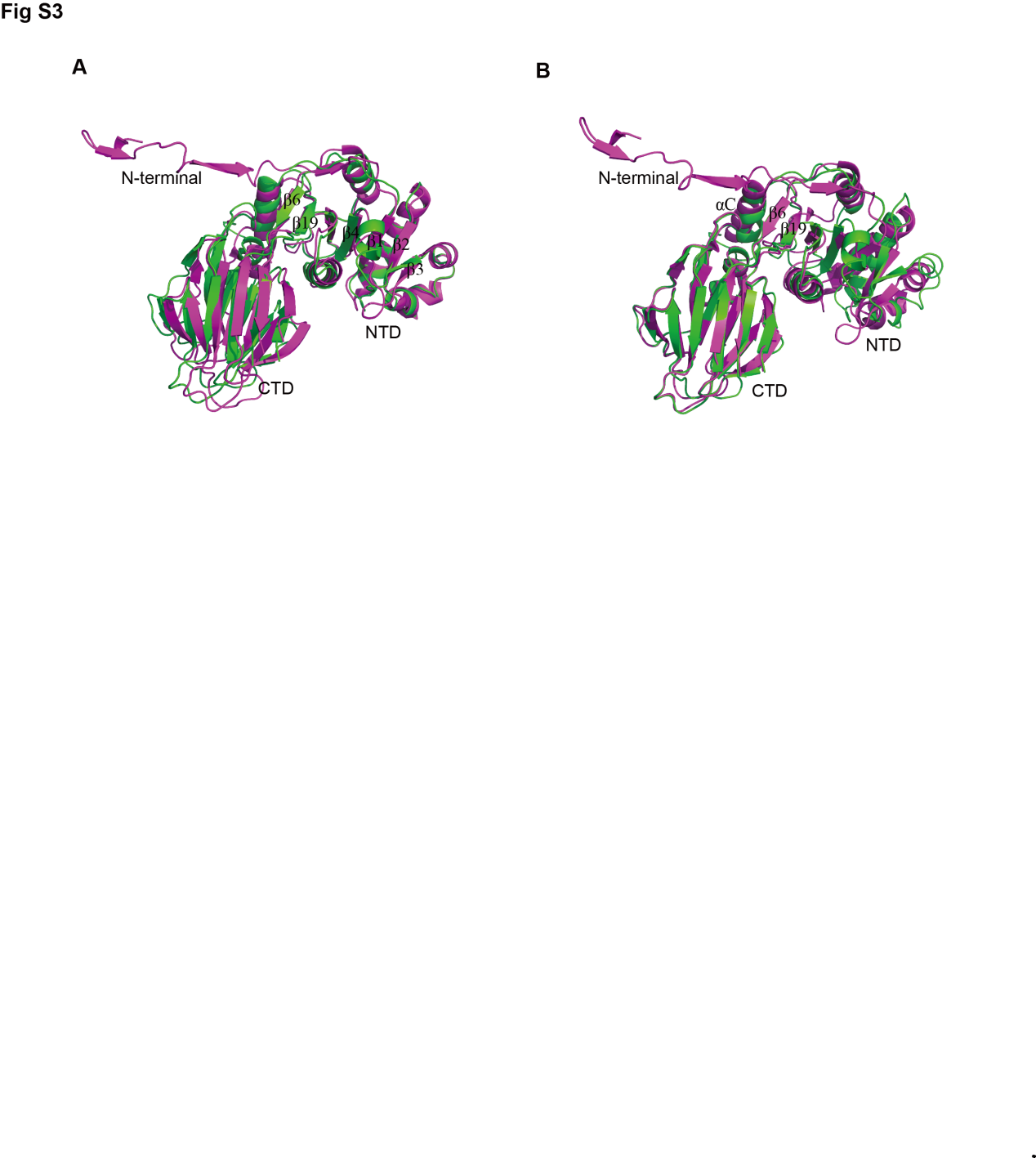
**

**Figure S3 NTD is more flexible than CTD.**

1. Alignment of the cryo-EM structure and crystal structure of NADK based on the NTD. NTD, CTD, the N-terminal, strands β1-β4, and the domain linker strands β6 and β19 are labeled.
2. Alignment of the cryo-EM structure and crystal structure of NADK based on the CTD. NTD, CTD, the N-terminal, and the C-terminal αC helix is labeled.

**
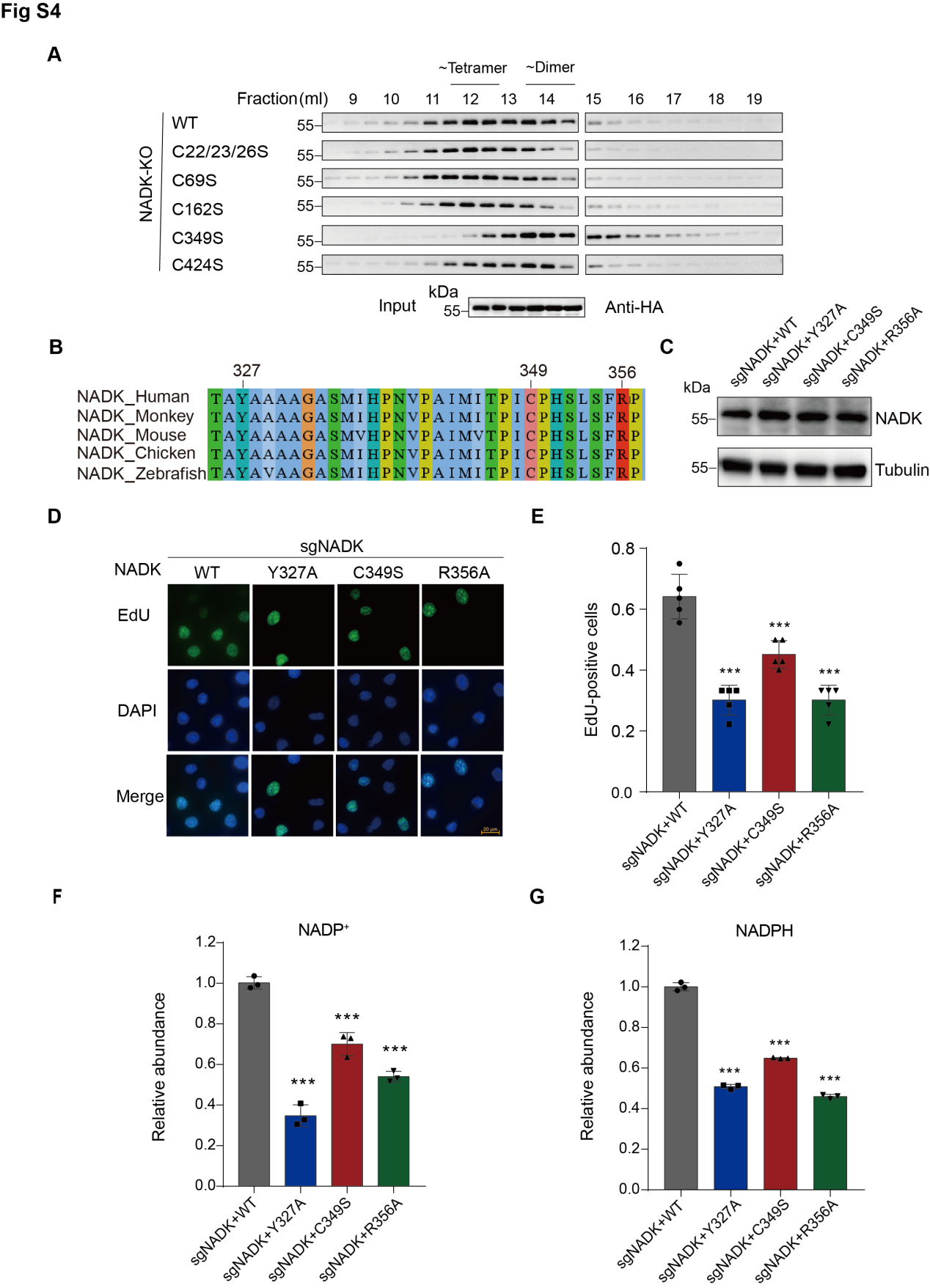
**

**Figure S4 - Disruption of NADK tetramer formation decreases NADK activity.**

A. The indicated NADK WT and cysteine mutant plasmids were introduced into HEK293T cells with endogenous NADK knocked out. HA-NADK was eluted in size exclusion fractions on the same column in identical settings. Indicated fractions of cell lysates were used for immunoblot analyses.

B. Sequence alignment of the amino acid that is involved in tetramer formation in NADK.

C. H520 cells with stable sgRNA-mediated knockout of NADK were stably reconstituted with NADK WT or indicated mutants and NADK protein level was detected by immunoblot analyses with anti-NADK antibody

D, E. The reconstituted H520 cells were labeled with 10 μM EdU for 30 min, and EdU-positive cells were examined by immunostaining (D) (n=5). EdU-positive cells are presented in (E). Scale bars: 20 μm.

F, G. Relative abundance of NADP^+^ (F) and NADPH (G) quantified from these reconstituted H520 cell lines (n=3).

Data information: In (D-G), data are presented as mean ± SD. **P* < 0.05. ***P* < 0.01. ****P* < 0.001 (One-way ANOVA).

**
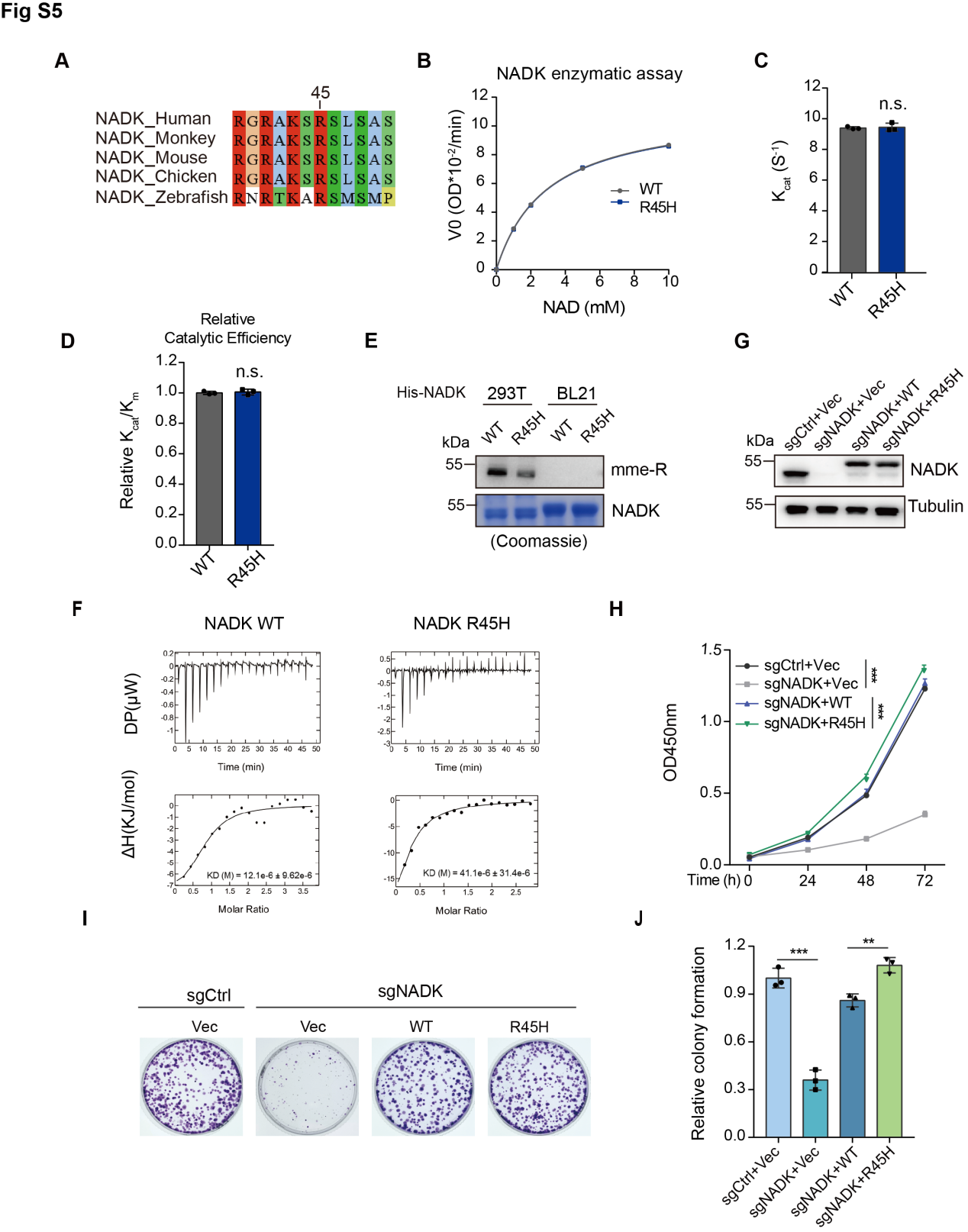
**

**Figure S5 R45H mutation increases NADK activity.**

1. Sequence alignment of NADK R45.

B-D. NADK enzymatic assay with purified NADK WT and R45H mutant proteins from *E. coli* BL21 strain (n=3). Michaelis-Menten (B), K_cat_ (C) and Relative catalytic efficiency (D) are presented.

E. NADK WT and R45H mutant proteins were purified from HEK293T cells or *E. coli* BL21 stain and NADK methylation level was detected by immunoblot analyses with anti-MME-R antibody.

F. ITC binding curves for NADK WT and R45H mutant proteins with substrate molecule NAD^+^. The Kd values, when measurable, are shown in the Figure, while non measurable values are indicated by NA.

G. The NADK knockout H520 cells were stably reconstituted with NADK WT or R45H mutant and NADK protein level was detected by immunoblot analyses with anti-NADK antibody.

H. Cell growth assay was performed by measuring OD450 from day1 to day4 (n=4).

I, J. Colony formation assay was performed to explore cell proliferation capacity (n=3). The cells were immobilized with formaldehyde and stained with crystal violet (I). Relative colony formation is presented in (J).

Data information: In (B-D, H-J), data are presented as mean ± SD. **P* < 0.05. ***P* < 0.01. ****P* < 0.001 (One-way ANOVA).


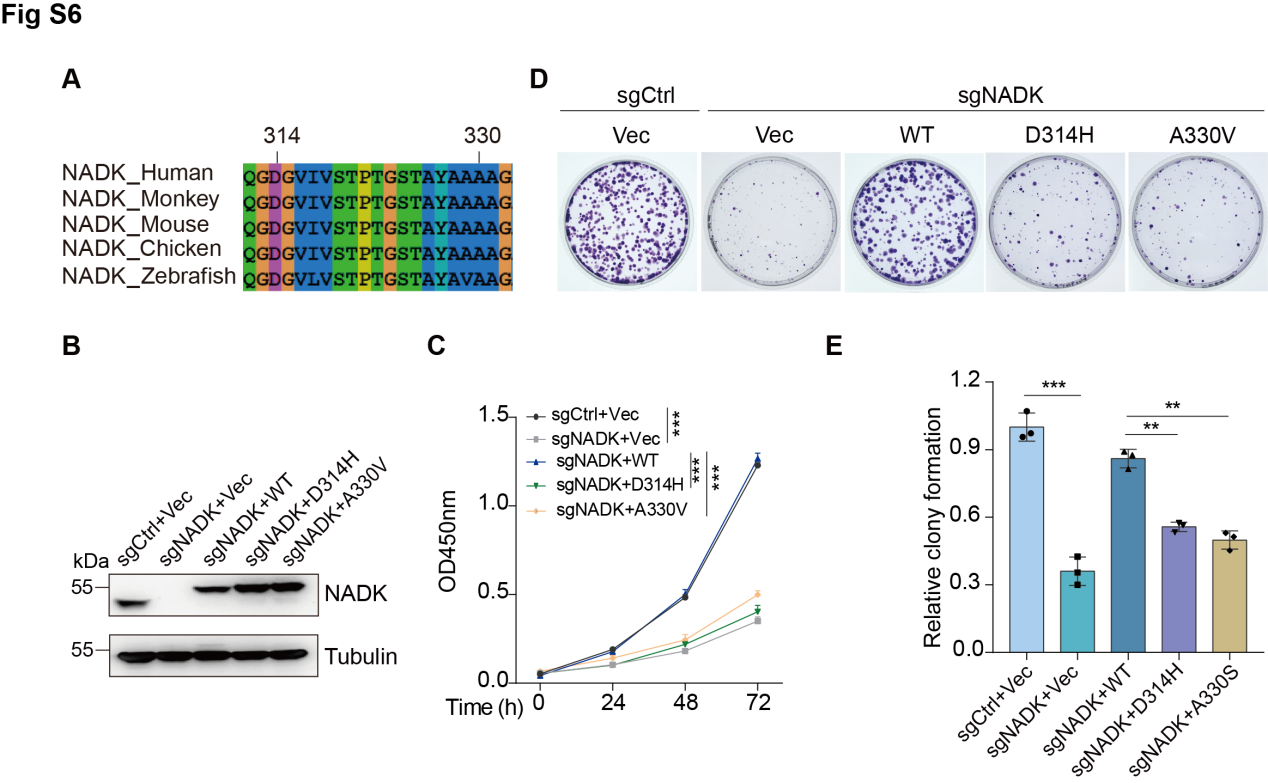


**Figure S6 NADK activity deficient mutants reduced the cell proliferation**

1. Sequence alignment of NADK D314 and A330.
2. The NADK knockout H520 cells were stably reconstituted with NADK WT or indicated mutants and NADK protein level was detected by immunoblot analyses with anti-NADK antibody.
3. Cell growth assay was performed by measuring OD450 from day1 to day4 (n=4).

D, E. Colony formation assay was performed to explore cell proliferation capacity (n=3). The cells were immobilized with formaldehyde and stained with crystal violet (D). Relative colony formation is presented in (E).

Data information: In (C-E), data are presented as mean ± SD. **P* < 0.05. ***P* < 0.01. ****P* < 0.001 (One-way ANOVA).
